# Seeing the full picture of brain development: an interview with Aparna Bhaduri on single-cell genomics and developmental neuroscience

**DOI:** 10.1038/s42003-021-02831-6

**Published:** 2021-11-19

## Abstract

Dr. Aparna Bhaduri is an Assistant Professor at the University of California, Los Angeles (UCLA). Dr. Bhaduri received her PhD in 2016 from Stanford University, and completed a post-doctoral research fellowship at the University of California, San Francisco, before starting her independent research career at UCLA. In this Q&A, Dr. Bhaduri tells us about her current work, the joys and challenges of starting a lab during a pandemic, and recent advances in developmental neuroscience.

**Figure:**
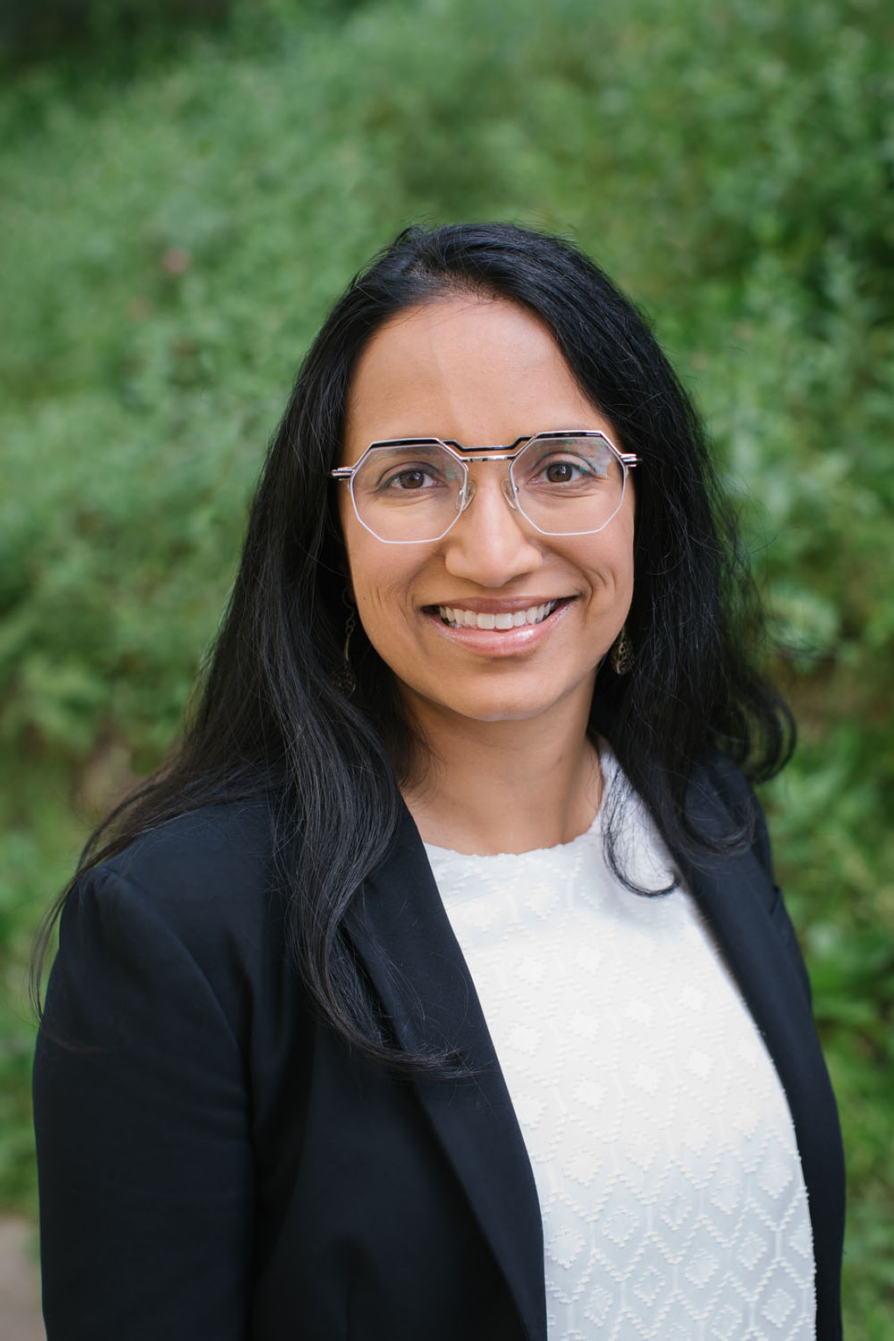
Aparna Bhaduri

Please tell us about your academic background and research interests.

I started my scientific career as an undergraduate at Rice University where I double-majored in Biochemistry and Cell Biology (BS) and Political Science (BA). Through college I had two main passions—working in the lab of Dr. Seiichi Matsuda on understanding the catalytic activity of oxidosqualene cyclases and competing on the university debate team. Eventually, science won out and I did my PhD at Stanford University in the lab of Dr. Paul Khavari, studying the interplay of epidermal differentiation and epithelial cancers. My training in the Khavari lab really set the stage for my future as I came to appreciate the value of human based model systems, I learned bioinformatics for the first time and was thrilled by the power of both wet lab experimentation and dry lab analysis, and I developed a fascination for how developmental programs are reactivated in cancer. Taking these lessons with me, I did my postdoctoral work in the lab of Dr. Arnold Kriegstein where I explored human cortical development and glioblastoma from the lens single-cell atlases of the developing human brain. This was an incredibly fun time to be in the genomics space, and to be studying the brain. My own lab at UCLA is studying how the complex cell fate transitions that we can characterize from these atlas-scale studies are actually regulated, and also how this impacts glioblastoma tumor heterogeneity and lineage.

Your doctorate focused on cancer genomics and differentiation. What inspired your move to more neuroscience research?

I cannot overstate how transformative my graduate research in the Khavari lab was for my personal scientific development. I entered graduate school very much assuming it was a mistake I had been admitted (I had accidentally written the wrong school name in my final essay submitted with the application!) and was terrified about the prospect of leading my own projects in a new field. I rotated in the Khavari lab because it had a lot of graduate students (10 on average during my tenure there) so I figured Dr. Khavari knew what he was doing. This was the absolute best choice I made because he and my mentors within the lab really supported me as I learned to analyze genomic data, failed miserably at countless Western blots, and pushed me to gain the confidence to bring new analyses and experimental techniques to our systems. Those six years taught me to be fearless when taking on new challenges, so when looking for a postdoc position, changing fields did not feel daunting. While I found the research on epithelial biology interesting, I have always gravitated towards the brain when reading popular science news and even in certain undergraduate classes. I think the human brain, specifically, is the most beautiful marvel of evolutionary engineering, and after graduate school I had the confidence to really pursue this fascination. It was fortuitous that the Kriegstein lab was performing so much single-cell RNA-sequencing and needed someone capable of performing this analysis, as my informatic skills motivated Dr. Kriegstein to overlook my lack of developmental neuroscience experience. I am thrilled to have made this choice—in my own lab I get to share this awe and wonder with my trainees every day and I think the experiments we have ongoing now using single-cell genomics and cortical organoids (stem cell-derived models of the developing human brain) are incredibly exciting!

How do you think single-cell omic approaches have changed the field of neurodevelopment, and what do you think is the most exciting application of these tools?

I think single-cell omic approaches have cracked open the opportunity to really understand how the brain emerges during development, as well as how it matures and changes during the lifespan. Prior to single-cell omics, we had genomics from averaged profiles of all brain cells or candidate by candidate interrogations in brain tissue. Honestly, it’s incredibly inspiring how these methodical assays really elucidated key concepts about brain development and very humbling to note that the most obvious cell type markers and transcriptional regulators were identified in this way. However, I do believe that single-cell omic approaches allow us to now see the full picture of neurodevelopment, including how co-expression networks and less well-characterized genes can really refine a cell’s ultimate fate. I think the most exciting application of these tools is really in human disease biology, where we are beginning to understand how small changes in these cell fates can have lasting impacts. I think this is especially true in glioblastoma where we can see how developmental cell populations are recapitulated in the cancer, but with some discrepancies. I think these discrepancies are the key to understanding vulnerabilities of the cancer and also to understand how certain genes are essential to normal neural stem cell fate specification.

What has it been like starting a lab (or interviewing for faculty positions) during the pandemic? Do you have any advice for other early-career researchers?

It has been interesting and challenging to finalize my faculty position and start the lab during the pandemic. Supply chain issues have forced us to be creatively resourceful at times, and the mitigation strategies such as low lab density early in the pandemic and continued masking means that we have learned how to be effective with hybrid in-person and remote work structures. Some of this has actually spurred positive innovation—we are lucky to live in beautiful Los Angeles so we have a lovely outdoor weekly lab meeting where those who choose to can take off their mask, and getting outside is actually really refreshing and fun. There are still some things that I am worried about: trainees have suffered immensely during the pandemic and I worry about their mental health being in graduate school or doing a postdoc during this bizarre time. Supply costs are skyrocketing, and although I don’t have children, I know many junior faculty are still struggling with pandemic related childcare issues, among other challenges. Starting a lab in general is nerve-wracking and I am just hoping that if and when the money runs out from our startups, institutions and the NIH will recognize the especially unique challenges junior faculty from my cohort have faced and will help out. I am always seeking community with other early-career researchers, and my advice to others would be to do the same as much as possible. So many things about this job can only be commiserated with others in the same position, and I have found these discussions to be motivating and often hilarious.

What is the most exciting part about being a new faculty member, and are there any traditions you would like to start in your lab?

The two most exciting things about being a new faculty member are the trainees and the ability to follow my own ideas. I have the absolute best job in the world. Every day, I get to work with enthusiastic, intelligent, creative people and watching them grow and learn is absolutely astonishing. It is also amazing that I am getting paid and funded to pursue the ideas in neurodevelopment and brain cancer that I find most compelling. We are also getting to the point where some of the trainees are building on these initial ideas, and it’s just so much fun to watch the lab blossom. I also am thrilled that the environment at UCLA is highly collaborative, and these collaborations are really enabling us to move our science forward at a faster rate. I am letting the lab take lead on potential traditions, but we have enjoyed some Bhaduri lab swag (water bottles), lab lunches, and various impromptu celebrations!

*This interview was conducted by Associate Editor George Inglis*.

